# The Significant Impact of Lost Species' Identity, Number and Abundance on Functional Structure of Alpine Meadow

**DOI:** 10.1002/ece3.72136

**Published:** 2025-10-15

**Authors:** Zhiyong Yang, Ci‐ren Qu‐zong, Yuan Zhang, Xine Li, Skalsang Gyal, Wei Mazhang, Ying Yang, Guotai Zhang, Cuo Se, Danzeng Quzhen, Jingting Mao, Chengwei Mu, Lan Wang, Shiping Wang, Tsechoe Dorji

**Affiliations:** ^1^ State Key Laboratory of Tibetan Plateau Earth System, Environment and Resources Institute of Tibetan Plateau Research, Chinese Academy of Sciences Beijing China; ^2^ Naqu Alpine Grassland Ecosystem National Field Scientific Observation and Research Station Naqu Xizang China; ^3^ School of Ecology and Environment Xizang University Lhasa Xizang China; ^4^ School of Geographical Sciences and Tourism Zhaotong University Zhaotong Yunnan China; ^5^ College of Animal Science and Technology Yangzhou University Yangzhou Jiangsu China

**Keywords:** alpine meadow, ecological niche, removal experiment, species loss

## Abstract

Biodiversity, the cornerstone of ecosystem functions and services, faces threats from anthropogenic climate change. However, it remains unclear how species loss, including abundance, number, and identity of lost species, would alter the functional structure of alpine meadow plant communities. Through a species removal experiment conducted in an alpine meadow of the central Tibetan Plateau, we found that removing common species typically altered the functional structure of plant communities. Changes in community functional structure were highly positively correlated with both the number of species removed and the degree of abundance loss. Beyond this, species removal treatments had significant direct effects on community functional structure, as revealed by a partial least squares path modeling approach. This indicates that the effects of species identity are independent of the number of species lost and the degree of abundance loss. Especially, only the removal of species including *Kobresia pygmaea* significantly reduced community abundance when fewer than three species were removed. This indicates that the species *Kobresia pygmaea* occupies an exclusive ecological niche and is irreplaceable in alpine meadows, suggesting that the consequences of climate change‐induced declines in *K. pygmaea* biomass may be underestimated. Moreover, ecological niche breadth rather than niche overlap played a primary indirect role in the pathway through which species loss influences functional structure. This indicates that harsh environmental filtering and species adaptation, rather than biotic interactions, dominate community assembly in alpine meadows. This study provided valuable insights for biodiversity conservation and adaptive management of alpine ecosystems.

## Graphical abstract

Beyond the effects of lost abundance and lost species number, the identity of lost species still has significant effects on the functional structure of alpine meadow plant communities. *Kobresia pygmaea* occupies an exclusive ecological niche and is irreplaceable in alpine meadows, suggesting that the consequences of climate change‐induced declines in *K. pygmaea* biomass may be underestimated. Ecological niche breadth rather than niche overlap played a primary indirect role in the pathway through which species loss influences functional structure. This indicates that harsh environmental filtering and species adaptation, rather than biotic interactions, dominate community assembly in alpine meadows.

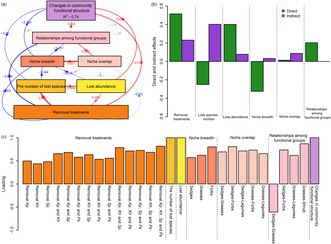

## 1 Introduction

Biodiversity underpins ecosystem processes and functions (del Río et al. 2016; Forrester and Bauhus 2016; Harrison et al. 2014; Li et al. 2014; Midgley 2012; van der Plas 2019). However, biodiversity has been under severe threat from global change and intensified human activities (Cahill et al. 2013; Niemandt and Greve 2016; Oliver and Morecroft 2014; Weiskopf et al. 2020). Species loss is occurring at an unprecedented rate (Butler et al. 2003; Ceballos et al. 2015), yet the consequences of species loss on ecosystem functional structure, especially the loss of common species in vulnerable ecosystems, remain largely unclear (Fu et al. 2021), which hinders our ability to develop and implement necessary conservation measures.

The alpine meadows dominated by *Kobresia pygmaea*, spanning approximately 450,000 km^2^ on Tibetan Plateau, are the largest pastoral alpine ecosystem on earth (Miehe et al. 2019). These meadows are highly sensitive to climate change and human activities, such as overgrazing and warming trends, which threaten their ecological integrity (Huang and Fu 2023; Shen et al. 2022; Wang et al. 2022; Wang, Xue, et al. 2023; Wei et al. 2023). Overgrazing, a prevalent issue, has led to the depletion of soil nutrients and water resources, exacerbating species loss in this fragile ecosystem (Cao et al. 2019; Mo 2020). Additionally, climate warming poses a significant threat, inducing rapid species loss in alpine meadows across the Tibetan Plateau (Dorji et al. 2018; Klein et al. 2004; Quan et al. 2021; Zhang et al. 2015; Zhao et al. 2024). This warming trend has particularly affected *K. pygmaea*, with previous studies indicating a significant decline in its population due to temperature increases and altered environmental conditions (Hopping et al. 2018; Jiang et al. 2017; Ma et al. 2017; Peng et al. 2017). In addition to *K. pygmaea* (Sedges), *Kobresia humilis* (Sedges), *Stipa purpurea* (Grasses), and *Potentilla saundersiana* (Forbs) are also common species with relatively high abundance in alpine meadows. The loss of these species would inevitably alter functional group structure in alpine meadow plant communities (Hypothesis 1). Plant functional groups classify species with similar traits into categories, simplifying community‐level processes and serving as a bridge between species and ecosystems (Lopez et al. 2021; Peng et al. 2022; Zhang et al. 2022). Evaluating the alterations in the structure of plant community functional groups due to species loss can aid in understanding the process by which species loss affects ecosystem function. Currently, studies simulating species loss typically focus on the responses of overall ecosystem attributes, with less attention paid to changes in the functional group structure of communities (Li et al. 2020; Rewcastle et al. 2022; Sundqvist et al. 2020; Wardle et al. 2020; White et al. 2020).

Moreover, species loss encompasses three dimensions: abundance, species number, and species identity (Abalos et al. 2014; Cesarz et al. 2013; Larsen et al. 2005; Ma et al. 2021). The greater the loss of abundance, the more severe the potential impact on the functional structure of the community (Hypothesis 2). However, the effect of losing a greater number of species should not be solely attributed to the loss of species abundance, as the loss of more species increases the likelihood of affecting multiple functional groups, thereby possibly leading to more substantial changes in the community's functional structure (Hypothesis 3). Furthermore, the impact of species identity itself should not be overlooked (Hypothesis 4). Species identity is determined by morphological, functional, and other characteristics (Laughlin 2024; Lefcheck et al. 2021), and different species, due to their unique traits, occupy distinct ecological niches (Buche et al. 2022; Carroll and MacDougall 2024). Species loss may alter the functional structure of the community by changing the niche breadth, overlap, and interactions between functional groups (Hypothesis 5). If the lost species occupies a unique ecological niche, then its loss cannot be fully compensated by any other species, and the niche breadth, overlap, and biotic interactions between functional groups will all undergo significant changes (Godoy et al. 2020; Sexton et al. 2017), ultimately leading to substantial shifts in the functional structure of the community (Hypothesis 6). If the ecological niche of the lost species is entirely encompassed by the niches of other species, then the species is completely redundant: if the loss of the species leads to a reduction in abundance that is compensated by species from other functional groups, its loss will not only reduce the niche breadth of its functional group but also may alter the niche overlap between functional groups, thereby changing the biotic interactions between them (Slade et al. 2017; Wang, Liu, et al. 2023), the functional structure will still be altered (Hypothesis 7); if the loss of its abundance can be compensated by species within the same functional group, resulting in no significant changes to the functional structure of the plant community (Hypothesis 8). Currently, studies evaluating the effects of species loss often fail to distinguish between these three aspects.

In an alpine meadow in central Tibetan Plateau, we conducted a foundational species removal experiment to address the following questions: (1) Does species loss significantly alter the functional structure of alpine meadow plant communities (Hypothesis 1)? (2) Does the impact of species loss on the functional structure of the plant community increase with the magnitude of the removed abundance (Hypothesis 2)? (3) Beyond the effects of abundance loss, do the number and identity of lost species still have significant effects on the functional structure of alpine meadow plant communities (Hypotheses 3 and 4)? (4) What roles do functional group niche breadth, overlap, and interactions between functional groups play in the pathway by which species loss influences the functional structure of the community (Hypothesis 5)? (5) How did these removal treatments alter the relative richness at the functional group and community levels (Hypotheses 6–8)?

## 2 Methods

### 2.1 Study Site

This removal experiment was conducted relying on Naqu Alpine Grassland Ecosystem National Field Scientific Observation and Research Station, located at Kema village, Luoma town, Naqu Prefecture, Tibet Autonomous Region, China (31.27° N, 92.09° E, 4500 m a.s.l) (Appendix S1: Figure S1) (Su et al. 2020). The experiment site is an alpine meadow under a typical alpine mountain climate, which is cold and dry enough to not allow trees to inhabit (Körner 2012; Miehe et al. 2019). In this region, the mean annual temperature is −1.2°C, and the mean annual precipitation is 430 mm. More than 80% of precipitation falls in summer from June to September (Ganjurjav et al. 2016; Suonan et al. 2024; Zhu et al. 2016). The short and cold growing season generally starts in May and lasts until September (Miehe et al. 2019; Zhu et al. 2016). The experiment site was previously grazed by yak but has been fenced for excluding grazing since 2013. The vegetation is dominated by *Kobresia pygmaea* and *Stipa purpurea*, accompanied by *Kobresia humilis*, *Potentilla saundersiana*, *Lancea tibetica*, and *Aster tataricus* .

### 2.2 Experimental Design

**FIGURE 1:**
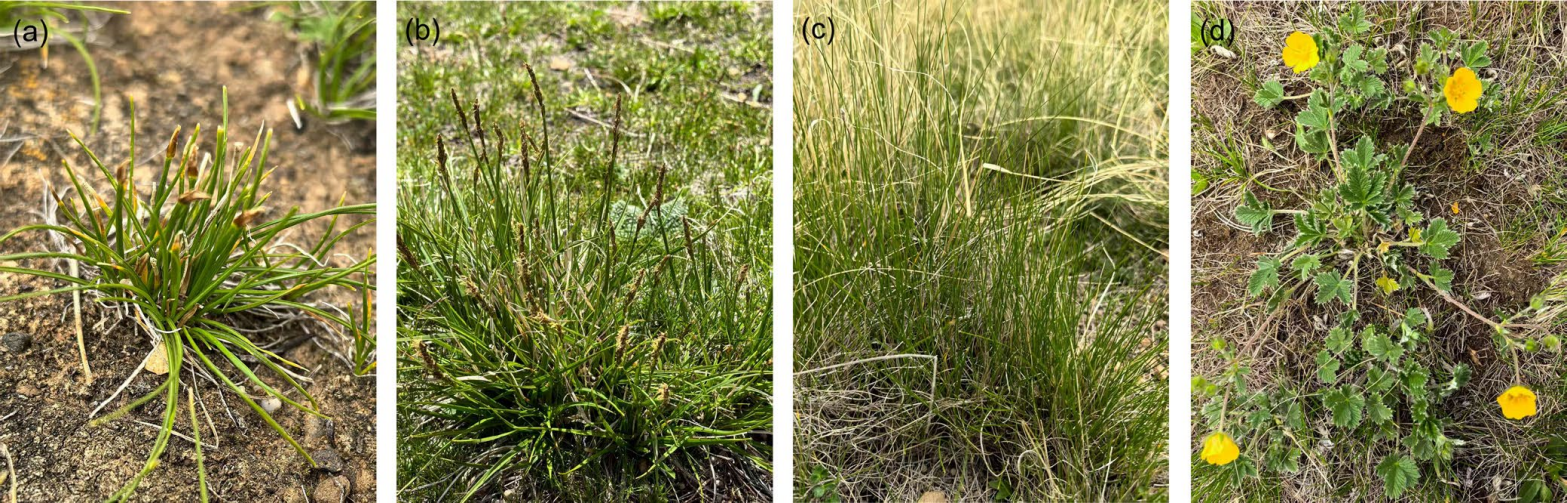
Organism photographs of four removed species. (a) *Kobresia pygmaea* (*Kp*); (b) *Kobresia humilis* (*Kh*); (c) *Stipa purpurea* (*Sp*); (d) *Potentilla saundersiana* (*Ps*).

**FIGURE 2:**
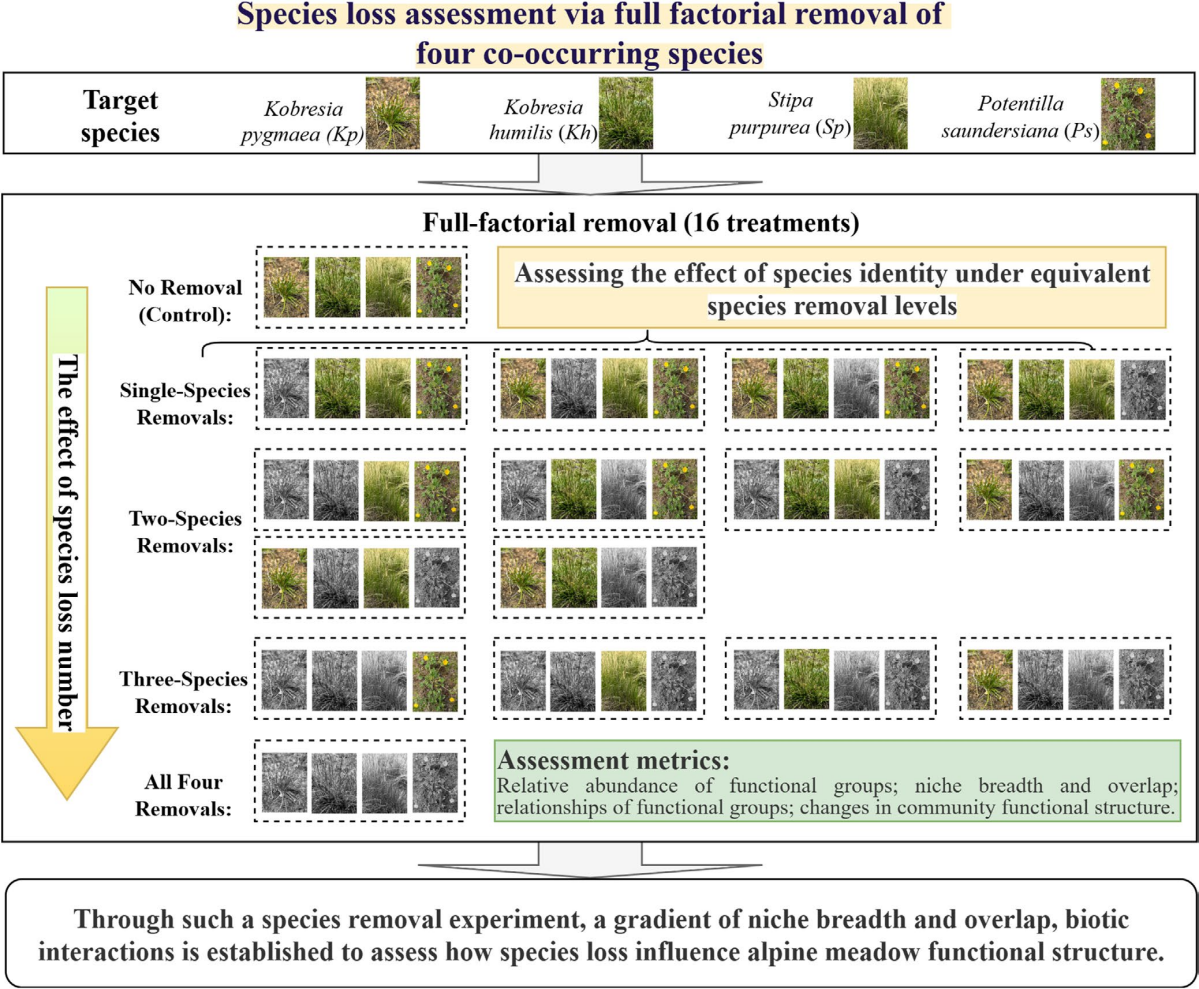
A conceptual diagram of the species removal experiment. Each dashed rectangle represents an experimental treatment. Green species images indicate species that remain (not removed), while black species images indicate species that have been removed.

This removal experiment was established in July 2013 following a randomized block design. Four common species, including *Kobresia pygmaea* (*Kp*), *Stipa purpurea* (*Sp*), *Kobresia humilis* (*Kh*), and *Potentilla saundersiana* (*Ps*), were selected for removal manipulations (Figure 1; Appendix S1: Figure S2). One to four species among them were randomly removed, forming a full factorial combination and a no‐removal control, consisting of 16 treatments (Figure 2). There were four replicates per treatment, and each plot was 1 m × 1 m with at least 2‐m buffer strips between plots, for a total of 64 plots. Since 2014, removals have been carried out in early July every year. To minimize disturbance to permanent plots, the removal of biomass in this study was limited to above‐ground tissues.

### 2.3 Community Monitoring

In early August every year, relative abundance for each species was investigated in each plot using the point intercept method (Kardol et al. 2018; Wardle et al. 2020). Specifically, each plot was evenly divided into 400 small squares, and the species intercepted by the point in the upper right corner of each square was recorded.

### 2.4 Statistical Analysis

Consideration of the initial disturbance effects of the removal manipulations (Wardle et al. 2020; Wardle and Zackrisson 2005), the first 5 years of data (2014–2018) was excluded from the data analyses.

Linear mixed‐effects models (restricted maximum likelihood [REML] estimation) were used to assess the effects of species removal, including both the number and identity of species lost, on plant community functional structure. Changes in plant community functional structure were calculated as the Bray dissimilarity index between the treatment and control plots based on the relative abundance of plant functional groups (sedges, grasses, forbs, legumes, and shrub). First, to evaluate the effect of the number of species removed, we included year and the number of removed species as fixed effects, with block as a random effect. Then, to assess the effect of species identity for each level of species loss, we grouped the data from treatments with the same number of species removed together with the control, and applied a similar model, treating year and species removal treatments as fixed effects and block as a random effect. After these data were standardized by *Z*‐score, the coefficients of these models were the effect sizes of these independent variables. Furthermore, the relation of these effect sizes with removal relative abundance from control plots was evaluated using a linear regression model. Similarly, the effects of species removal on the relative abundance at the functional group and the community levels were evaluated.

A species' niche breadth refers to the range of environments it can inhabit (Gaston et al. 1997). It is important to note that the base ecological niche, the range of environments in which a species can live and reproduce (Futuyma and Moreno 1988), is shaped by evolutionary history and phylogeny (Sexton et al. 2017), so here we only explored changes in realistic ecological niche—the set of abiotic and biotic environments a species occupies (Futuyma and Moreno 1988)—due to species loss. Niche overlap refers to the similarity of environments or resources used by the species (Giustino et al. 2018; Ma et al. 2019; Wang, Liu, et al. 2023). Ecology niche breadth and overlap were calculated using the Levins index and the Pianka index, respectively, for each treatment. The specific calculation formulas for niche breadth and overlap are as follows (Genitsaris et al. 2020; Wang, Liu, et al. 2023):

$$B_{i}=1/\sum_{j=1}^{n}P_{\mathrm{ij}}^{2},$$

$$Q_{\mathrm{ik}}=\sum P_{\mathrm{ij}}P_{\mathrm{kj}}/\sqrt{\sum P_{\mathrm{ij}}^{2}\sum P_{\mathrm{kj}}^{2}},$$

where *B*_*i*_ indicates the niche breadth of functional group *i*; *O*_*ik*_ indicates the niche overlap value between functional groups *i* and *k*; and *P*_*ij*_ and *P*_*kj*_ indicate the ratio of relative abundance of functional groups *i* and *k* at resource *j* to the total abundance for all plots. Each plot indicates a resource bit and n is the plot number.

Moreover, to assess the impact of removals on biotic interactions between plant functional groups, relationships among sedges, grasses, forbs, legumes, and shrubs were calculated for each treatment using partial correlation (Wang, Liu, et al. 2023; Yoon et al. 2019). In the partial correlation analysis, the relative abundance of each plant functional group was included as a variable. For the relationship between sedges and grasses, the partial correlation coefficient between the relative abundance of sedges and grasses was calculated, setting the relative abundance of other plant functional groups as the controlling variables. The relationships between other functional groups were determined similarly, respectively.

Finally, to understand how the loss of common species influences plant community functional structure, a partial least squares path modeling (PLS‐PM) was performed, incorporating factors such as species removal treatments, the number of lost species, the abundance that was eliminated, niche breadth and overlap, relationships between functional groups, and changes in plant community functional structure. PLS‐PM is a causal modeling technique that combines principal components analysis, canonical correlation analysis, and multiple regression (Meng et al. 2024), which decomposes both direct and indirect effects of each factor on community functional structure. To ensure the model quality, observed variables with loadings less than 0.4 were removed, as these variables were considered to contribute insufficiently to the model.

## 3 Results

**FIGURE 3:**
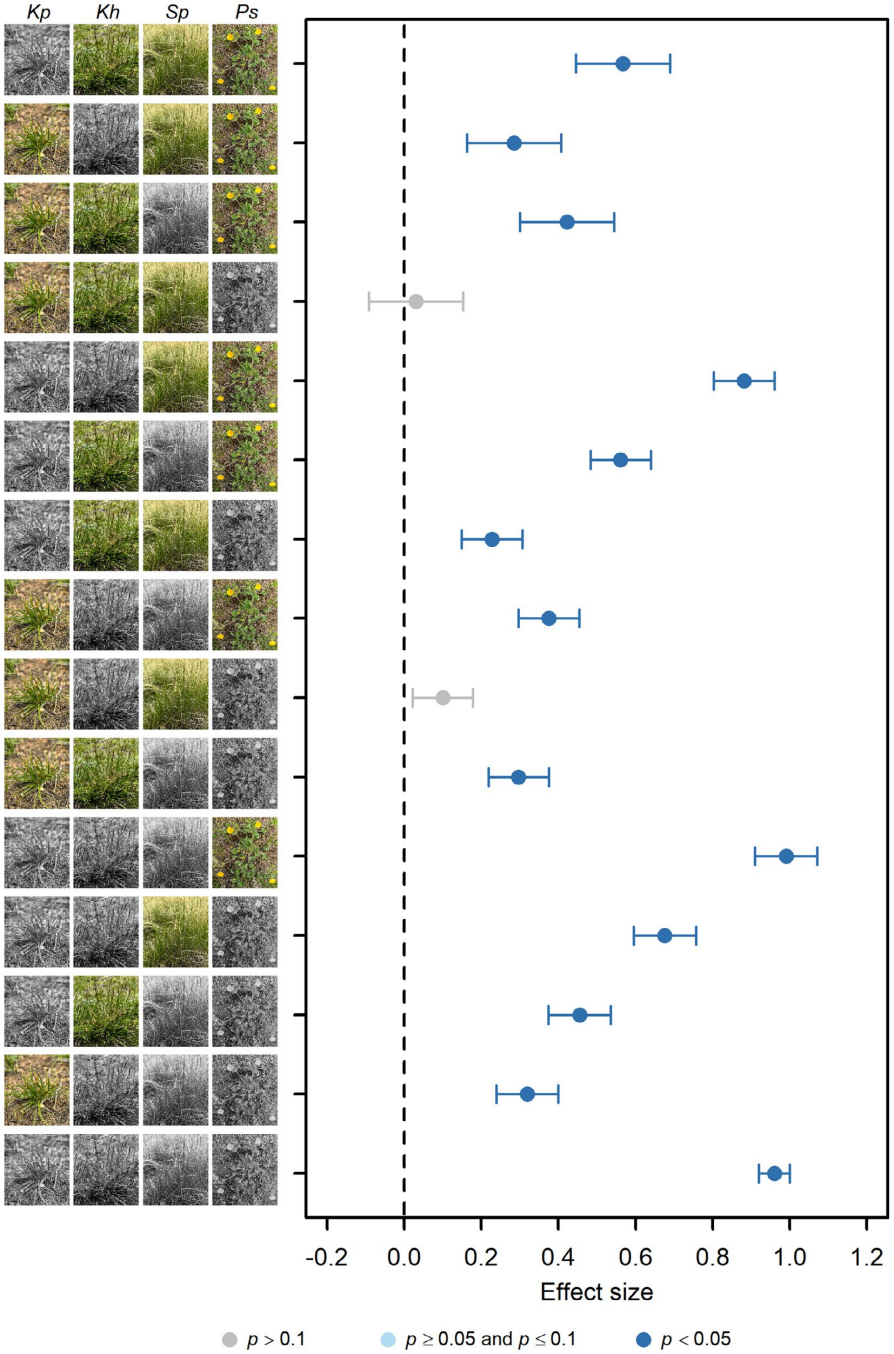
The effects of removing species identity on functional structure of plant community when the number of species removed is held constant, with effect sizes represented by coefficients from linear mixed models, where year and species removal treatments were treated as fixed effects and block as a random effect, using standardized data by *Z*‐score. The error bar represents one standard error. Green species images indicate species that remain (not removed), while black species images indicate species that have been removed.

Species loss generally significantly alters the functional structure of plant communities (Hypothesis 1), as evidenced by the significant increase in Bray dissimilarity between treatment and control groups, compared to the average level of dissimilarity among control plots, with the exception of the treatments where only *Ps* was removed and where both *Kh* and *Ps* were removed simultaneously (Figure 3; Appendix S1: Tables S1–S4). Overall, the greater the number of species removed, the more pronounced the changes in the functional structure of the community (Figure 3; Appendix S1: Table S5). When the number of species removed is the same, there are significant differences in the effects of removing different species identities on the community functional structure (Figure 3). Removing *Kp* has the strongest effect, followed by removing *Sp*, then *Kh*, and finally removing *Ps*, which has the weakest effect.

**FIGURE 4:**
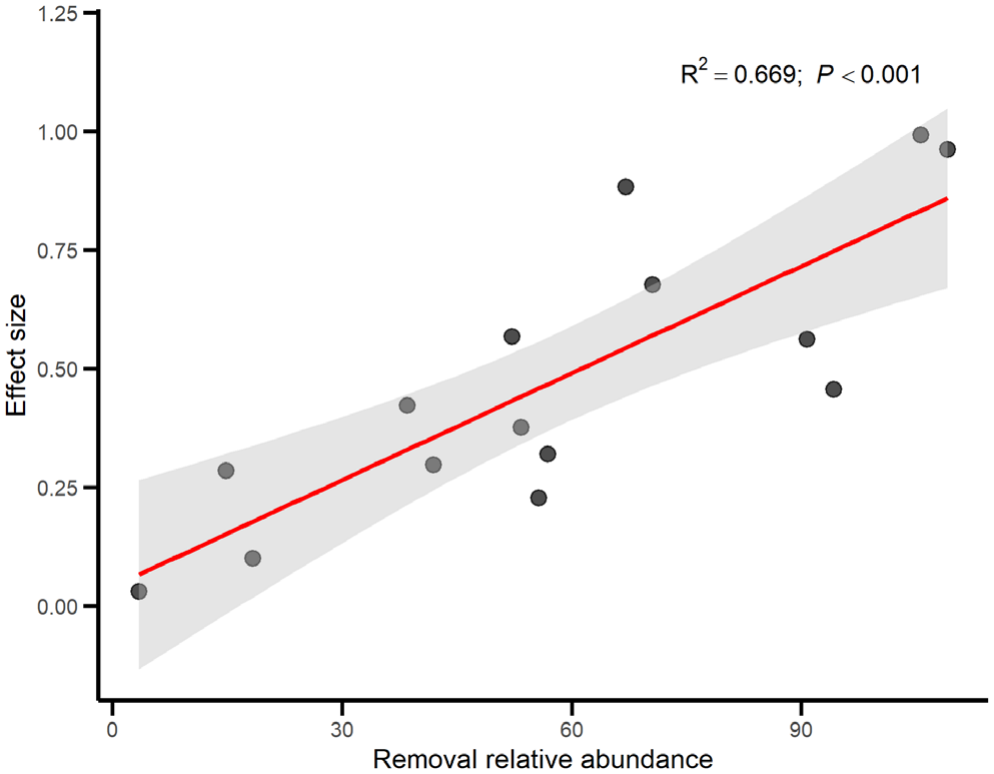
The relationships between effect sizes of removal treatments on functional structure of plant community and removal relative abundance. The red line represents the fitted line of the linear regression model. The gray shading indicates the 95% confidence interval.

Moreover, the effect size of the removal treatment is significantly positively correlated with the relative abundance of the removed species, with a goodness of fit as high as 0.669 (Figure 4) (Hypothesis 2).

**FIGURE 5:**
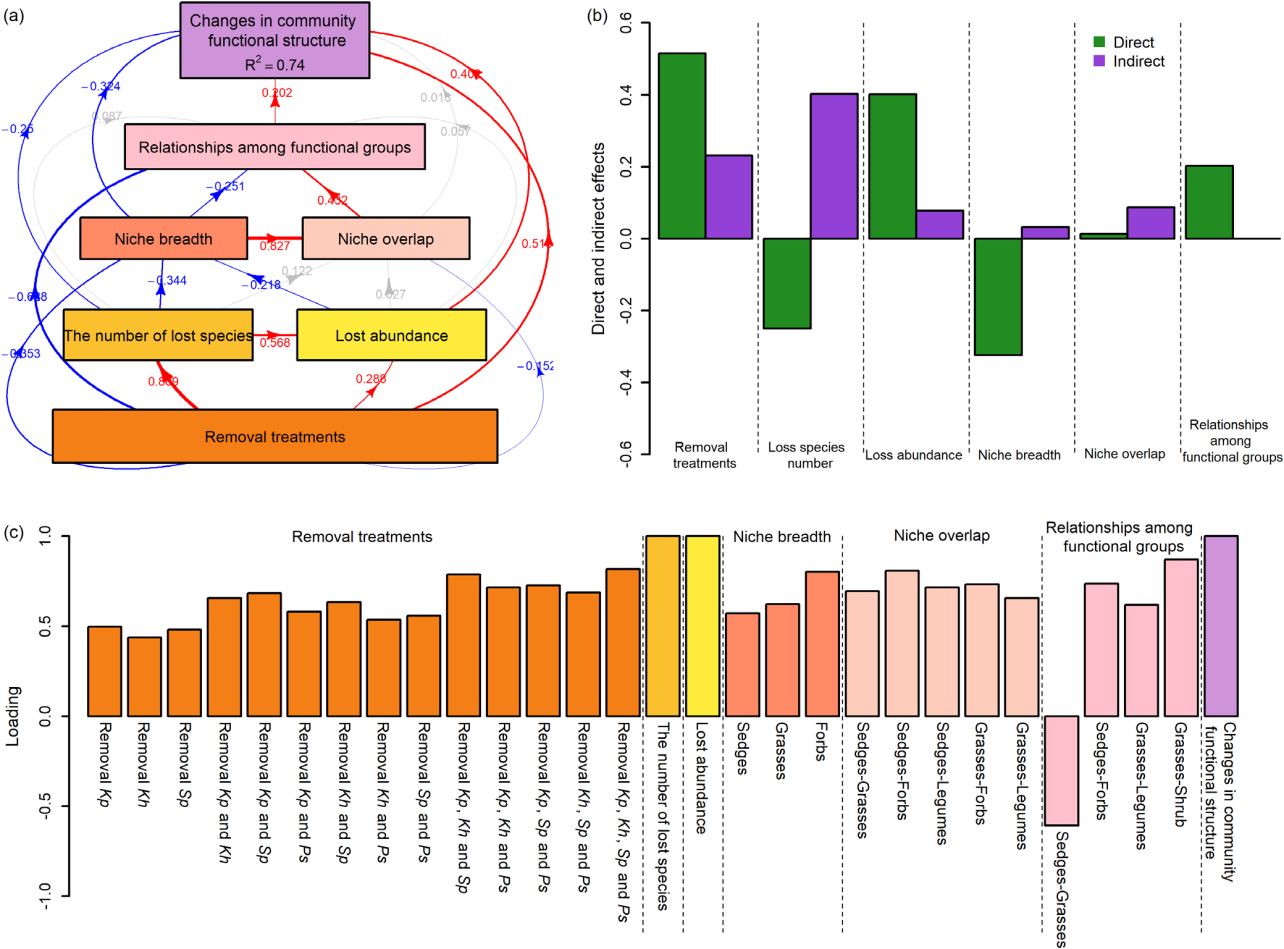
(a) The pathways of species removal effects on community biomass by the PLS‐PM analysis. Red and blue lines represent significantly positive and negative effects, respectively, while gray lines indicate no significant effect. (b) The direct and indirect effects of removal treatments, loss species number, loss abundance, niche breadth and overlap, and relationships among functional groups on community functional structure. (c) The loading of observed variables on latent variables.

In the PLS‐PM path analysis, even after considering the effect of lost abundance, the loss of species number still has a significant direct effect on the community's functional structure (Figure 5a) (Hypothesis 3); however, this direct effect is negative, despite the overall effect being positive (Figure 5b). Similarly, after accounting for the effects of both species loss number and abundance, the removal treatment still has a significant direct positive effect on the community's functional structure (Figure 5a), suggesting that the role of species identity itself persists and that the direct effect outweighs the indirect effect (Figure 5b) (Hypothesis 4). Moreover, the removal treatment, the number of species lost, and the relative abundance loss all directly and significantly reduce the niche width, particularly for sedges, grasses, and forbs, while the removal treatment also directly and significantly reduces niche overlap and functional group interactions, except for the interactions between sedges and grasses (Figure 5a,c). Niche width and overlap both significantly influence the functional structure of the community through the interactions between functional groups (Figure 5a) (Hypothesis 5). Additionally, niche overlap has no direct effect on the functional structure of the community, but niche width has a significant direct effect, and the direct effect is greater than the indirect effect (Figure 5a,b).

**FIGURE 6:**
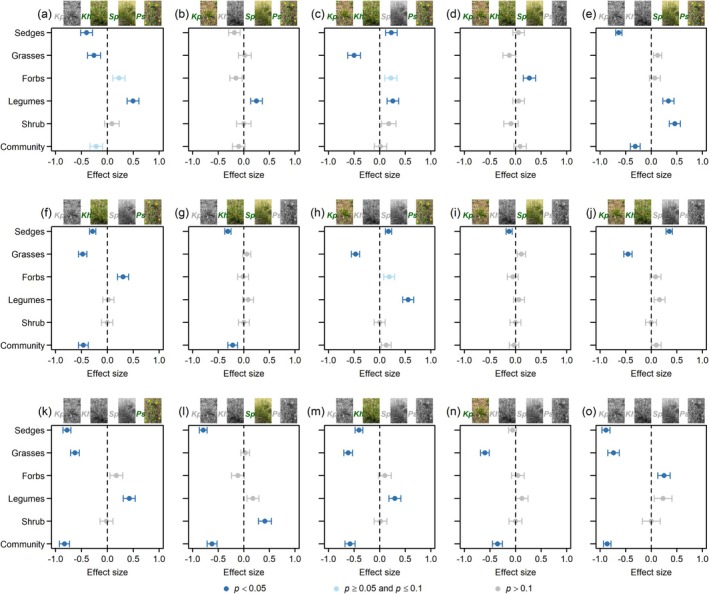
The effects of removing species identity on the relative abundance at the functional group and the community levels. The effect sizes were calculated as coefficients from linear mixed models, where year and species removal treatments were treated as fixed effects and block as a random effect, using standardized data by *Z*‐score. The error bar represents one standard error. Panels (a–d) show the removal of one species; (e–j) show the removal of two species; (k–n) show the removal of three species; (o) shows the removal of all four species. In each panel, green species images indicate species that remain (not removed), while black species images indicate species that have been removed.

When a single species is removed (Appendix S1: Table S6), only the removal of *Kp* marginally significantly (*p* < 0.1) reduces the relative abundance at the community level (Figure 6a) (Hypothesis 6). When only *Kh* is removed, although the relative abundance at the community level does not change significantly, the relative abundance of legumes significantly (*p* < 0.05) increases (Figure 6b). Similarly, when only *Sp* is removed, the relative abundance at the community level does not change significantly, but the relative abundance of grasses significantly decreases, while the relative abundance of sedges, legumes, and other forbs significantly or marginally significantly increases (Figure 6c) (Hypothesis 7). When only *Ps* is removed, there are no significant changes in the relative abundance of other functional groups or at the community level (Figure 6d) (Hypothesis 8). When more species are removed, the relative abundance of functional groups generally undergoes drastic changes, with the exception of the *Kh* and *Ps* removal treatments; especially when the removed species included *Kp* or more than two species are removed, the relative abundance at the community level significantly decreases (Figure 6e–o; Appendix S1: Tables S7–S9) (Hypothesis 6).

## 4 Discussion

It is widely known that species diversity promotes ecosystem functioning, due to the distinct roles different species play in ecological processes based on their functional traits (Dietrich et al. 2024; Duffy et al. 2017; Hector and Bagchi 2007; Isbell et al. 2018; van der Plas 2019). However, under the impacts of climate change and human activities, species diversity is undergoing unprecedented stress (Ceballos et al. 2015; Fu et al. 2021; Niemandt and Greve 2016; Weiskopf et al. 2020). Therefore, the question arises: How do species loss, including the identity, number, and abundance of lost species, alter the functional structure of ecosystems? While many studies on the removal of plant functional groups have been conducted (Elumeeva et al. 2018; Li et al. 2018; Wardle et al. 2020; Zhou et al. 2019), research focusing on the impact of species removal on the functional group structure of plant communities remains limited. We conducted a common species removal experiment in the alpine meadows of the central Tibetan Plateau and found that species removal generally altered the functional group structure of alpine meadow plant communities; the identity, number, and abundance of lost species all had significant direct effects on community functional structure; and changes in niche breadth played an important role in this process. This study is the first to assess the impact of species loss on the functional structure of alpine meadow plant communities dominated by *Kobresia pygmaea*, enhancing our understanding of how alpine grassland ecosystems respond to the loss of common species.

As different functional groups play distinct roles in ecosystem processes, changes in functional group structure may significantly impact overall ecosystem functioning, including nutrient cycling, photosynthetic capacity, and aboveground biomass (Li et al. 2020; Melendez Gonzalez et al. 2019; Pan et al. 2016; Ward et al. 2009; Wardle et al. 2020). For instance, McLaren (2009) observed significant effects of functional group removal on soil nutrients such as total nitrogen, nitrate (${\mathrm{NO}}_{3}^{-}$), phosphorus (P), and sulfur (S) in a relatively dry grassland near Kluane Lake, Yukon, Canada. Additionally, the decomposition of litter was influenced by the removal of specific functional groups in this grassland. Furthermore, in an ombrotrophic peatland in northern England, UK, it was found that the identity of plant functional groups affects short‐term carbon fluxes (Ward et al. 2009). Similarly, Li et al. (2018) demonstrated that the 4‐year removal of dominant grasses significantly decreased the total aboveground biomass in a subalpine meadow on the Tibetan Plateau. Our results showed that most of the species removal treatments significantly altered the functional group structure of the alpine meadow plant community (Figure 3), as expected under Hypothesis 1. This suggests the functional vulnerability of the alpine meadow ecosystem dominated by *K. pygmaea* under species loss scenarios.

Species richness is a key factor influencing ecosystem functions, generally considered to enhance ecosystem functionality and stability due to complementarity effects and increased functional redundancy (Barry et al. 2019; Biggs et al. 2020; Dietrich et al. 2024; Eisenhauer et al. 2023; Fetzer et al. 2015; Jops and O'Dwyer 2023; van der Plas 2019; Wardle and Palmer 2016; Zhou et al. 2019). However, the role of species identity might be even more critical (Cesarz et al. 2013; Lefcheck et al. 2021; Ma et al. 2021; Petruzzella et al. 2020; Slade et al. 2017). Yet, studies detecting the role of species identity in grassland ecosystems remain limited (Abalos et al. 2014; Hines and Eisenhauer 2021; Larsen et al. 2005). For instance, it has been found that plant species identity surpasses species richness as a key driver of N_2_O emissions from grassland (Abalos et al. 2014). Similarly, we observed that the impact of species identity on the functional structure of alpine meadow communities was greater than that of the number of lost species (Figure 5b), likely due to differences in species traits. Changes in species identity and number inevitably lead to alterations in abundance. Some studies have shown that the loss of abundance resulting from species extinctions also contributes to functional loss, but they did not quantify the distinctions between the identity, number, and abundance of extinct species (Larsen et al. 2005). We also found a highly positive correlation between the lost abundance and the functional structure of the community (Hypothesis 2). Furthermore, we observed that in the alpine meadows of the Tibetan Plateau, in addition to the abundance of lost species, the identity and number of lost species also had significant effects on the functional structure of the community (Hypotheses 3 and 4). In a northern Canadian grassland, Melendez Gonzalez et al. (2019) suggested that ecosystem properties are determined by plant biomass, regardless of species composition. Aside from abundance, other traits of species, such as plant stoichiometric characteristics, metabolism, and photosynthetic rates, may also influence the functional structure of alpine meadow plant communities. Interestingly, in the path analysis, the direct effect of the number of lost species on the functional structure of the community was negative, meaning that the more species lost, the smaller the change in the functional structure of the community. The overall positive effect of the number of lost species on changes in the functional structure of the community (Appendix S1: Table S5; Figure 3) was indirectly realized through changes in niche breadth and interactions between functional groups (Figure 5). This may imply that the direct effect of the number of lost species itself is merely a random statistical effect—the more species are lost, the more balanced the changes in each functional group are likely to be.

According to ecological niche theory, niche breadth and overlap determine species performance and biotic interactions (Godoy et al. 2020; Hutchinson 1957; Ma et al. 2019), and thus community attributes (Haberstroh and Werner 2022; Wang, Liu, et al. 2023). As Hypothesis 5 predicts, species loss significantly altered niche breadth and overlap, thereby changing the interactions between functional groups, ultimately affecting the functional structure of the community (Figure 5). Moreover, the effect of niche overlap was relatively weak, with the effect of niche breadth being much greater than that of niche overlap. This indicates that harsh environmental filtering and species adaptation, rather than biotic interactions, drive community assembly in alpine meadows. This is consistent with the findings of Liu et al. (2023), who argued that habitat filtration was the main driving force of community assembly at high altitudes (4000 and 4500 m). Under the harsh environmental conditions of the Tibetan Plateau, species that are most adapted to the background environment should occupy unique ecological niches, with the largest niche breadth and abundance. The loss of such species inevitably leads to a decline in overall community abundance. In this study, we found only the removal of species including *Kp* significantly reduced community biomass when the number of removed species was fewer than two (Figure 6). This indicates that the foundation species *Kp* occupies an exclusive ecological niche and is irreplaceable in alpine meadows. Its loss necessarily alters ecological niches and biological interactions (Appendix S1: Tables S10–S12), leading to profound changes in community functional structure (Figures 5 and 6) (Hypothesis 6). For other species removal treatments, significant decreases in community abundance occurred only when more than three species were removed. Interestingly, the removal effects of *Kh*, *Sp*, or *Ps* alone on community abundance were not significant. However, joint removals of *Kh*, *Sp*, or *Ps* with *Kp* exhibited more pronounced statistical effects compared to single‐species removal of *Kp* (Figure 6). This suggests that niches of *Kh*, *Sp*, and *Ps* could be mostly contained within the base ecological niche of *Kp*. This is consistent with the known experience that there is a positive asymptotic relationship between ecosystem functions and biodiversity, which implies some species are redundant (Hector and Bagchi 2007). The results of removing *Sp* fully align with Hypothesis 7 (Figure 6c). Removing only *Sp*, although it did not significantly alter community abundance, the abundance loss caused by *Sp* removal could not be compensated by other species within the same functional group, but instead, it was compensated by species from other functional groups. This ultimately led to significant changes in the functional structure of the community. On the other hand, the results of removing *Ps* fully align with Hypothesis 8 (Figure 6d). The loss of abundance caused by *Ps* removal was fully compensated by other species within the same functional group and even overcompensated. Consequently, removing only *Ps* did not significantly alter the functional structure of the alpine meadow plant community (Figures 3 and 5). These findings demonstrate that the impact of species loss on community functional structure varies among species, further emphasizing the role of species identity. Moreover, most of the changes in the relative abundance of sedges, grasses, legumes, and shrubs with removal manipulations can be explained by ecological niche processes, particularly niche breadth, but niche only explained part of the variation in relative abundance of forbs (Appendix S1: Figure S3). The forbs were composed mainly of rare species (Appendix S1: Figure S1), which had very low population growth rates constrained by the harsh environment of alpine meadows (Liu et al. 2023; Yang et al. 2012; Zhu et al. 2020). Maybe, the portion of forbs abundance change not explained by ecological niche was from neutral processes (Hubbell 2001, 2005, 2006; Matthews and Whittaker 2014). A similar pattern was also found by Bell (2000) and Magurran and Henderson (2003), where the abundance distributions of rare species were shaped by neutral processes, such as stochastic birth, death, and immigration. Somewhere in between niche and neutral processes, one possible explanation was that removal manipulations could change population growth rates and fitness differentiation (Cadotte and Tucker 2017; Chu et al. 2017). Theoretical studies based on process modeling suggested that community structure and dynamics under severe stress environments were mainly controlled by stochastic processes (Xiao et al. 2013; Yang et al. 2015). However, our results indicate that deterministic processes (ecological niche differentiation and fitness differentiation) and stochastic processes operate simultaneously in the alpine plant community.

Biotic interactions are important processes that determine plant community assembly (Garcia‐Callejas et al. 2021; Wisz et al. 2013; Yeakel et al. 2020), thus modulating the effects of species diversity on ecosystem functions and their stability (Eisenhauer et al. 2019; Grossiord 2020; Haberstroh and Werner 2022). In this study, we use partial correlation analysis to quantify the interactions between plant functional groups and found that facilitative interactions were common in alpine meadows (Appendix S1: Table S12), which was in line with the stress gradient hypothesis and previous empirical studies (Cavieres et al. 2014; Dominguez et al. 2002; Lima et al. 2022; Lortie and Callaway 2005; Wang et al. 2008). Cushion species generally appear to have a nurse effect on other species in harsh alpine ecosystems (Cavieres et al. 2014; Verdu et al. 2021). However, the sedges dominated by *Kp* with a characteristic root mat were usually negatively correlated with grasses in this study (Appendix S1: Table S12). This may be due to the enhanced competitive pressure on sedges from population growth of grasses following grazing exclusion in the enclosed grassland (Dorji et al. 2018; Wang et al. 2022). This was supported by the result that the niche overlap between sedges and grasses contributed more to the relative abundance of sedges than grasses (Appendix S1: Figure S3). Moreover, some limitations for inferring biotic interactions based on partial correlation analysis must be recognized. First, random changes in community structure (e.g., environmental disturbances or neutral processes) may lead to non‐biotic correlations in species abundance. Second, partial correlation only reflects statistical association and cannot be directly used to infer causation. Third, partial correlation coefficient inherently assumes symmetric interaction between variables; however, real ecological interactions often exhibit asymmetry.

## 5 Conclusions

Biodiversity, the cornerstone of ecosystem functions and services, is facing serious threats in the context of global change, especially in fragile ecosystems such as the Tibetan Plateau (Weiskopf et al. 2020). Effective biodiversity conservation has already been urgent (Oliver and Morecroft 2014). Through the removal experiment, we found removing common species typically altered the functional group structure of alpine meadow plant communities. Changes in community functional structure were highly positively correlated with both the number of species removed and the degree of abundance loss. Beyond these, species removal treatments had significant direct effects on community functional structure, which implies that the effects of species identity are independent of the number of species lost and the degree of abundance loss. Especially, our results indicate that species *Kp* occupied a unique ecological niche, and its contribution to ecosystem functional structure was irreplaceable by other species in alpine meadows. This implies that the consequences of climate change‐induced declines in *Kp* biomass may be underestimated (Jiang et al. 2017; Ma et al. 2017; Peng et al. 2017). Furthermore, in the indirect pathways through which species loss influences functional structure, it is primarily the niche breadth rather than niche overlap that plays a mediating role. This suggests that harsh environmental filtering and species adaptation, rather than biotic interactions, are the main driving forces behind community assembly in alpine meadows. Additionally, while facilitative interactions are generally present in alpine meadows, this is not always the case, as evidenced by the negative correlation between sedges and grasses. This study can provide a theoretical basis for biodiversity conservation and adaptive management of alpine ecosystems in the context of global change. For example, prioritize monitoring and protecting *K. pygmaea* (*Kp*) population. Implement managed grazing practices to suppress grasses growth, thereby reducing competitive pressure on sedges, particularly *Kp*.

## Author Contributions

**Zhiyong Yang:** conceptualization (equal), data curation (equal), formal analysis (lead), writing – original draft (lead). **Ci‐ren Qu‐zong:** data curation (lead). **Yuan Zhang:** formal analysis (equal), writing – original draft (equal). **Xine Li:** conceptualization (equal), data curation (equal), writing – original draft (equal). **Skalsang Gyal:** writing – original draft (equal). **Wei Mazhang:** data curation (equal). **Ying Yang:** data curation (equal). **Guotai Zhang:** data curation (equal). **Cuo Se:** data curation (equal). **Danzeng Quzhen:** data curation (equal). **Jingting Mao:** data curation (equal). **Chengwei Mu:** data curation (equal). **Lan Wang:** data curation (equal). **Shiping Wang:** conceptualization (equal), funding acquisition (equal). **Tsechoe Dorji:** conceptualization (lead), funding acquisition (lead), writing – original draft (equal).

## Conflicts of Interest

The authors declare no conflicts of interest.

## Supporting information


**Appendix S1:** Supporting Information.

**Appendix S2:** Supporting Information.

**Appendix S3:** Supporting Information.

**Appendix S4:** Supporting Information.

**Appendix S5:** Supporting Information.

## Data Availability

The data and codes producing the findings of this study have been uploaded as [Link], [Link], [Link], [Link] for review and publication.
