# Supplementary material for: The Significant Impact of Lost Species' Identity, Number and Abundance on Functional Structure of Alpine Meadow
Source: Ecol Evol. 2025 Oct 15;15(10):e72136. doi: 10.1002/ece3.72136 (PMC12522019; doi:10.1002/ece3.72136)
Supplement: Supplementary file 1 — Appendix S1: Supporting Information. [file ECE3-15-e72136-s001.docx]

Metadata File for Species Removal Experiment in Alpine Meadow Ecosystem, Naqu, Tibet Autonomous Region, China

Zhiyong Yang^1,2a^, Ci-ren Qu-zong^1,2,3a^, Yuan Zhang^1,3,4^, Xine Li^5^, Skalsang Gyal^1,3^, Wei Mazhang^1,3^, Ying Yang^1^, Guotai Zhang^1,3^, Cuo Se^1,3^, Danzeng Quzhen^1,3^, Jingting Mao^1^, Chengwei Mu^1,3^, Lan Wang^1,3^, Shiping Wang^1,2,3*^, Tsechoe Dorji^1,2,3*^

^1^State Key Laboratory of Tibetan Plateau Earth System, Environment and Resources, Institute of Tibetan Plateau Research, Chinese Academy of Sciences, Lhasa, China

^2^Naqu Alpine Grassland Ecosystem National Field Scientific Observation and Research Station, Tibet, China

^3^School of Ecology and Environment, Tibet University, Lhasa, China

^4^School of Geographical Sciences and Tourism, Zhaotong University, Zhaotong, China

^5^College of Animal Science and Technology, Yangzhou University, Jiangsu, China

*Author for correspondence: Tsechoe Dorji, Email: [tsechoedorji@itpcas.ac.cn](mailto:tsechoedorji@itpcas.ac.cn); Shiping Wang, Email: wangsp@itpcas.ac.cn

^a^ZY and CQ contributed equally and should be considered joint first author

**Study Description**

This study investigates the effects of species removal on alpine meadow ecosystem. The experiment was conducted at the Naqu Alpine Grassland Ecosystem National Field Scientific Observation and Research Station in Kema village, Luoma town, Naqu Prefecture, Tibet Autonomous Region, China. The site is located at an elevation of 4500 m a.s.l. (31.27° N, 92.09° E). The region experiences a typical alpine climate, with a mean annual temperature of −1.2 °C and mean annual precipitation of 430 mm, with more than 80% of precipitation falling between June and September. The growing season is short and cold, lasting from May to September.

The experiment was established in July 2013 using a randomized block design. Four dominant species—*Kobresia pygmaea* (*Kp*), *Stipa purpurea* (*Sp*), *Kobresia humilis* (Kh), and *Potentilla* *saundersiana* (*Ps*)—were selected for removal manipulations. Treatments included all possible combinations of species removal (full factorial design), resulting in 16 treatments, including a no-removal control. Each treatment was replicated four times, with each plot measuring 1 m × 1 m and buffered by at least 2 m between plots, totaling 64 plots. Since 2014, removals have been carried out in early July every year. Biomass removal was limited to above-ground tissue to minimize disturbance to the plots.

**Temporal Coverage**

2019-2022 (The first five years of data were excluded from the data analyses to minimize the initial disturbance effects of the removal manipulations).

**Data Collection**

Each plot was evenly divided into 400 small squares, and the species intercepted by the point in the upper right corner of each square were recorded in early August every year.

**‌Data File Naming and Usage:‌**

1. **‌Main Data File:**‌

- ‌File Name:‌ Data.csv
- ‌Contents:‌
- year (Year)
- Plot (Plot number)
- Remove (Removal treatment code)
- Repeat (Replicate number)
- Sp.code (Species code)
- Species (Latin name of species)
- Frequency (Frequency of occurrence)

1. **‌Removal Treatment File:**‌

- ‌File Name:‌ Removal manipulations.csv
- ‌Contents:‌
- Treat (Treatment code)
- Remove.Kp (Whether *Kobresia pygmaea* is removed, 1=yes, 0=no)
- Remove.Kh (Whether *Kobresia humilis* is removed, 1=yes, 0=no)
- Remove.Sp (Whether *Stipa purpurea* is removed, 1=yes, 0=no)
- Remove.Ps (Whether *Potentilla saundersiana* is removed, 1=yes, 0=no)

‌Notes:

‌Binary coding (1/0):‌

1 indicates the species ‌was removed‌ in the treatment.

0 indicates the species ‌was not removed

1. **‌Species Code File:**‌

- ‌File Name:‌ Species code.csv
- ‌Contents:‌
- Code (Species code)
- Name (Latin name of species)
- Group (Plant functional group)
